# Feminist approaches to Anorexia Nervosa: a qualitative study of a treatment group

**DOI:** 10.1186/s40337-017-0166-y

**Published:** 2017-11-13

**Authors:** Su Holmes, Sarah Drake, Kelsey Odgers, Jon Wilson

**Affiliations:** 1 Department of FTM, University of East Anglia, Norwich, NR4 7TJ UK; 2 School of HSC, University of East Anglia, Norwich, NR4 7TJ UK; 3 Norfolk and Suffolk Foundation Trust, 80 St Stephens Road, Norwich, NR1 3RE UK

**Keywords:** Treatment, Anorexia nervosa, Group, Gender, Feminism, Sociocultural, Qualitative

## Abstract

**Background:**

Eating disorders (EDs) are now often approached as biopsychosocial problems. But it has been suggested by scholars interested in sociocultural factors that all is not equal within this biospsychosocial framework, with the ‘social’ aspects of the equation relegated to secondary factors within ED treatment contexts. Although sociocultural influences are well-established as risk factors for EDs, the exploration of whether or how such perspectives are useful *in treatment* has been little explored. In responding to this context, this article seeks to discuss and evaluate a 10 week closed group intervention based on feminist approaches to EDs at a residential eating disorder clinic in the East of England.

**Methods:**

The data was collected via one-to-one qualitative interviews and then analysed using thematic discourse analysis.

**Results:**

The participants suggested that the groups were helpful in enabling them to situate their problem within a broader cultural and group context, that they could operate as a form of ‘protection’ from ideologies regarding femininity, and that a focus on the societal contexts for EDs could potentially reduce feelings of self-blame. At the same time, the research pointed to the complexities of participants considering societal rather than individualised explanations for their problems, whilst it also confronted the implications of ambivalent responses toward feminism.

**Conclusions:**

Highly visible sociocultural factors in EDs – such as gender - may often be overlooked in ED clinical contexts. Although based on limited data, this research raises questions about the marginalisation of sociocultural factors in treatment, and the benefits and challenges including the latter may involve.

## Plain English summary

Eating disorders (EDs) are now often understood as being caused by a range of factors, whether these are biological, psychological or social. But it has been suggested that societal dimensions of EDs are often marginalised within treatment settings. In responding to these claims, this article seeks to discuss and evaluate a group which ran for 10 weeks at a residential ED clinic in the East of England. The groups were loosely based on feminist approaches to EDs (which see social constructions such as gender as central to the development of eating problems), and placed particular emphasis on the relationships between EDs and cultural constructions of femininity. The seven participants involved in the study suggested that situating their eating problems in social and cultural contexts was helpful, whilst the results also indicate the difficulties and challenges of approaching EDs within social (rather than individual) terms. Nevertheless, given that the demographic of ED patients remains predominantly female, the study raises questions about why the relationship between EDs and femininity is often overlooked in contemporary treatment.

### Ethical information

This study received ethical approval by the General research and Ethics Committee at the University of East Anglia (3 March, 2016, Chair of the General Research Ethics Committee, Professor Peter Kitson), and Research and Development approval from Norfolk and Suffolk NHS Foundation Trust (#15 199,028, 8 June 2016).

## Background

### Feminist approaches to anorexia nervosa: A qualitative study of a treatment group

Eating disorders (EDs) are now often approached as biopsychosocial problems, because they are widely recognised as multifactorial in origin [1]. As such, contemporary ED treatment often takes a multi-dimensional approach that moves beyond a focus on weight and food. For example, guidelines set out by the National Institute for Clinical Excellence (NICE) advocate the inclusion of ‘psychosocial’ elements (thus recognising the psychological *and* social factors that influence mental health) alongside the management of physical symptoms [2]. However, it has been suggested that there is a substantial and unwarranted imbalance within this biopsychosocial framework, with the ‘social’ aspects of the equation relegated to secondary or facilitating factors within ED treatment contexts [3–5]. In its extensive review of treatments for Anorexia Nervosa (AN), for example, the NICE guidelines offer no suggestion as to how to address the ‘psychosocial’ elements they outline - simply suggesting that they pertain to ‘weight and shape’ - and such perspectives are not even mentioned in the discussion of Bulimia Nervosa (BN), Binge Eating Disorder (BED) or Other Specified Feeding and Eating Disorder (OSFED). This is indicative of the fact that although sociocultural perspectives on EDs represent a visible area of study and have a significant empirical foundation [1, 6], very little has been written about them in the context of ED *treatment.* The reasons for this are complex and multifaceted, and can only be understood in relation to the sociocultural approaches themselves and their intellectual, epistemological and clinical status.

The interest in exploring sociocultural perspectives on EDs has emerged from the overlapping fields of sociocultural and feminist approaches to eating and body distress. Sociocultural perspectives have primarily focused on the idealisation of thinness in women [6] and the stigmatization of body fat in Western cultures. In demonstrating that the idealization of thinness in women *and* the prevalence of AN and BN increased during the last century [1], sociocultural approaches suggest that exposure to Western ideals of appearance generates body dissatisfaction and dieting behaviours which elevate the risk for ED development. In responding to the significance of gender as a risk factor for eating problems, preventative efforts have thus commonly targeted the internalisation of the thin ideal and wider body dissatisfaction, and the most successful interventions have been seen to reduce ‘thinness expectancies’ [1] and disordered eating practices in populations of girls and young women [7]. There is also empirical evidence to suggest that gendered sociocultural ‘pressures to be thin’ – as shaped by peers, family and media use - can be a maintenance factor in EDs [8–10] (and it would of course be strange if some of the factors that led to the ED were somehow no longer relevant once the eating problem was established). The same is true of family attitudes toward weight and food regulation [9], which themselves are often shaped by gendered roles and expectations [11]. In this regard, whilst here has been far more sociocultural research on gender as a risk rather than maintenance factor for EDs, the inclusion of gender-focused interventions within ED treatment contexts is warranted – a perspective reinforced by the feminist approaches discussed below.

As the other key area of work offering sociocultural perspectives on EDs, feminist approaches have sought to situate EDs in relation to the wider social expectations surrounding Western femininity, ranging from gendered discourses on appetite, sexuality, economic power to social roles [11–13]. In this regard, and in common with wider principles in feminist therapy and philosophy, they refuse to treat women as ‘encapsulated individuals, separate from… society and its structures’ [14] – thus rejecting what are seen as the individualising (and pathologising) discourses of biomedical perspectives. In terms of what the feminist approaches to EDs see as ‘culture’, the significance of the media in propagating a slender ideal has certainly been recognised in feminist research on EDs [3, 11]. However, later feminist work also been wary of over-emphasising ‘the inscriptive power of cultural images of thinness’, and thus the characterisation of EDs as ‘body image’ problems [15], suggesting that this may miss the complex meanings of eating/body distress as they unfold within wider inequities of gendered power [5]. In discussing qualitative interviews and larger clinical group studies in which girls/women talk about their experience of an ED, feminist work has emphasised how disordered eating may not necessarily be motivated by the drive for pursuit of thinness or any ‘distortion’ of body image, but rather by wider experiences of ‘restricted agency’ that are structurally gendered [16].

In this regard, feminist work has provided qualitative evidence about how EDs emerge from, and are maintained by, cultural constructions of femininity [17, 4, 12, 14, 13]. In so doing, such research has argued that eating/body distress should be situated within the discourses and practices of *normative* femininities - thus critiquing the medical construction of the problems as eating ‘disorders’. In this regard, feminist conceptions of EDs reflect and contribute to wider strategies in feminist therapies which seek to normalise and value womens’ experiences (whilst critiquing the very existence of these as ‘natural’) [14]. For example, feminist work on EDs has explored the cultural conflation between women’s eating and sexual desire, situating eating/body distress in relation to the ways in which women are called upon to exert greater regulation of ‘appetite’, both in relation to food intake and sexual desire [11, 3, 12, 17]. Alternatively, and in relation to AN, feminist scholarship has theorised the body as a site upon which contradictory discourses on femininity might play out: thinness and starvation are seen as rendering femininity small, weak and fragile, whilst the emaciated body has been read as a form of corporeal resistance - the rejection of feminine subjectivity through an escape into a childlike, boyish or ‘degendered’ form [3, 12]. In this regard, feminist approaches to EDs do not distinguish between gender as a ‘risk’ or ‘maintenance’ factor: they see the aetiology, symptoms and the very nature of EDs as inextricably imbricated within discursive constructions of Western femininity [15].

However, although many of the early feminist interventions which emerged in the 1980s were developed from within treatment contexts by women who were feminist therapists or counsellors [18, 11], it has been suggested that such approaches may find limited application in current clinical environments, both in terms of how EDs are conceptualised [5] and according to extant evidence of treatment experience [4]. Holmes suggests for example that even when cultural discourses surrounding femininity are perceived by the person to be of high significance, they may be marginalised or ignored within ED treatment [4]. For some scholars, the suggestion is that sociocultural understandings of EDs have never been adequately integrated into mainstream treatment. So in 1997 (and looking back over at least 20 years of feminist scholarship in the field), Katzman and Sing observed how the integration of sociocultural conceptions of EDs into ‘diagnostic formulations and treatment plans [for EDs] has lagged noticeably. As a result, a “respectful nod” is offered to culture while an undue emphasis on individual pathology has persisted’ [5]. As this suggests, there has been (at best) a tendency to pay lip service to sociocultural influences in the context of ED treatment in ways which render them contextual and/or peripheral. Sampson’s notion of accommodation vs transformation is useful here. As he suggests, the concept of ‘adding on’ is said to ‘be accommodative rather than transformative…because the new element is not seen to be constitutive of the phenomenon of interest…Rather, as the very words suggest, the new is simply added on to the old without fundamentally *changing* the old [our emphasis]’ [19]. In terms of how the biopsychosocial equation may be translated into ED treatment, this thus leaves the existing power of biomedical discourses intact. The reasons for changing treatment paradigms are multifaceted and complex. But it may be the case that the decreased visibility of the feminist perspectives has been accelerated by a neoliberalist climate that reduces the experience into constructions of mental illness, [6] as well as a context in which dominant ideas about evidence-based practice that prioritise research evidence that can be clearly manualised, measured and quantified [20].

Finally, although feminist approaches are critical of the ways in which the sociocultural aspects of EDs are often reduced to body image in both popular and medical discourse, it is important to acknowledge that it is through this concept that sociocultural issues may find *some* expression in contemporary ED treatment. Body image work - which can be variously cognitive or psychoeducational - necessarily takes in social and cultural contexts in so far as notions of the ‘ideal body’ are ‘socially shared’ rather than ‘the unique and idiosyncratic production of the individual…’ [21]. However, although there is a growing evidence base for body image interventions [22], it has also been suggested that such work may be marginalised in treatment contexts [23]. In addition, the extent to which body image work really incorporates a focus on societal contexts can be variable, and the concept may often be treated as an ‘individual property best examined at the individual level’ [21].

In summary, the sociocultural and feminist approaches do not deny that EDs may incorporate psychological or biological factors, but they see sociocultural dynamics (in particular gender ideologies) as core etiological features [6], and this distinguishes them from biomedical paradigms. But although there is a ‘substantial empirical foundation’ for the significance of sociocultural factors within the aetiology and maintenance of EDs [6], such issues may be marginalised within current ED treatment practices. This has in turn led to a context in which there is little work testing the value and efficacy of such approaches within clinical environments. As such, this article seeks to discuss and evaluate a 10-week closed group intervention for a group of seven female patients at a residential eating disorder clinic based on feminist approaches to EDs. In so doing, the research seeks to explore two key questions: To what extent were a series of groups on the relationship (s) between EDs and cultural constructions of femininity seen as useful and beneficial to the participants as part of their treatment? How did they respond to a perspective which sees EDs as primarily cultural, rather than individual, manifestations, and what were the implications of this for how they conceptualised their eating/body distress, and the possibilities of recovery?

## Method

### Sample and context of the group

Ethical approval by the authors’ educational institution together with local research governance approval was gained prior to the setting up of a group intervention, which took place August–November 2016. The research was conducted in a private in-patient facility in the East of England which, while able to treat a range of eating problems, primarily admits patients with a diagnosis of AN. Despite its private independent status, the clinic admits almost exclusively NHS patients, so it is required to meet specific NHS protocols and standards in the treatment it provides. The facility has capacity for 10 in-patients over the age of 18 (and it does not admit patients who are detained under the Mental Health Act) [24], and the vast majority of the residents (and staff) are female. As is now common in UK in-patient settings, treatment incorporates psychiatric, therapeutic and dietary interventions, and the focus is on a multi-dimensional approach which moves beyond a singular focus on weight and food. The weekly schedule involves one-to-one therapy, a team meeting with the patient’s consultant, and numerous group therapies on themes such as recovery, photography, music, yoga, creative writing and body image.

### Group characteristics and context

The new group intervention was facilitated by the first two authors of this article. The in-patient site was selected because it was local to the University and the only one in the county where the authors are based. In addition, the second author offers individual and group therapy interventions there on a regular basis in her capacity as a senior occupational therapist. The group met once a week for 1 h for a duration of 10 weeks. All patients were asked to sign a consent form agreeing to the terms of the study (its use as anonymised data in research) if they were willing to take part, and the participant information forms explained that the groups would focus on some of the sociocultural contexts of eating disorders, including constructions of gender. After distributing participant information forms and offering to answer any questions in person or via email, the facilitators returned 1 week later to collect the signed consent forms and begin the group. All of the patients (*N* = 7) who were currently resident in the clinic took part, with ages ranging from 19 to 51 (mean age = 26). All had a primary diagnosis of AN, all were female, and all but one participant – who identified her ethnicity as mixed race – defined their ethnic identity as white British. Although it is acknowledged here that sexual orientation may impact upon how women negotiate cultural constructions of femininity, the study did not collect data on how the participants identified in terms of sexual orientation for ethical reasons (acknowledging that this may be a sensitive issue).

The group operated as a closed group, meaning that no new patients could join the sessions once their run had commenced. The research was assessing the cumulative effects of the groups with 1 week building upon the next, and closed groups have been seen as offering the researcher a clearer sense of individual or group change [25]. In addition, the decision was made not to audio-record the groups themselves. The facilitators were concerned that this might inhibit open discussion, and the intention was to analyse the participants’ individual responses to the groups as a whole, rather than detail and document the dynamics of each particular session.

### Philosophy of the group

The research that exists on group treatment for EDs focuses more on BN than AN, and there is a notable lack of research evaluating group therapies for the latter [26]. Nevertheless, group treatments for AN have become a common component of both in and out-patient care, and benefits of group therapy are now recognised as communal support, discussion of shared experience, group therapeutic alliance, personal growth and increased self-esteem [27]. More specifically, group work was chosen as the focus for this research because exploring sociocultural aspects of EDs – in this case the cultural construction of femininity - necessarily involves considering and evaluating *shared* understandings of social constructs which extend beyond the experience of any one individual. In this regard, it is fully recognised here that sociocultural factors in EDs are not limited to gender, nor to girls/women, and that femininity is in any case embedded in intersectional discourses of ethnicity, class, sexuality and age. However, femininity as a category was the focus for this study because it remains the most visible aspect of EDs to be discussed in social and cultural terms, and as the discussion above shows, there is thus a considerable body of conceptual and empirical evidence supporting the significance of this relationship.

As with feminist therapies more widely, feminist perspectives on EDs have drawn on a wide range of approaches. Nevertheless, common strands have been a recognition of the role that social oppressions play in creating and maintaining eating and body distress, a sensitivity to power in therapeutic/treatment contexts, an emphasis on women’s strengths and collectivity, and a commitment to empower women to challenge and critique the structures which may have dis-embodied and otherwise repressed them [28]. In particular, the focus of the group was shaped by the suggestion that it may be worth reframing body image as *the* site of sociocultural significance in EDs, and weekly topics moved across what ‘culture’ might mean in relation to EDs; gendered constructions of appetite; cultural expectations surrounding female emotion and anger; ‘reading’ the starved body in relation to cultural prescriptions of femininity; to the gendered dynamics of ‘healthy’ eating/living and fitness cultures (see Table 1). In so doing, media forms were regularly used in the group - from television adverts, Disney films, press articles, image bank photography to social media - as a means to stimulate debate about the particular issue being explored.

**Table 1:** The group ran for 10 weeks and was facilitated by the first two named authors of the article. Each week, the participants were given an outline sheet that indicated what would be covered in the group the following week; the key questions being asked of the topic; and details of the media examples that would be used as case studies

Week, topic and indicative questions	Materials
1. The role of society and culture in shaping EDs What do we think causes EDs? How can we think about the relationship between society and EDs, and how do such perspectives relate to arguments that EDs are more about the individual, or biology or genetics? How do we feel about these explanations (as they relate to ‘us’)? Are different EDs positioned in different ways in these debates (i.e. is AN seen as more ‘media-induced’ than other EDs?)	Press articles on the relationship between EDs and the media (e.g. EDs increasing due to the rise of social media).
2. Appetite, gender and culture How do we receive messages and ideas about ‘appetite’ in society? What kinds of meaning and values are attached to ‘indulging’/abstaining from food/appetite? To what extent do cultural constructions of appetite and eating differ between girls/boys, men/women? How are cultural constructions of food appetite implicitly or explicitly linked to wider constructions of ‘appetite’, such as those associated with sexual desire, or ambition?	TV adverts for diet/ low fat products which feature representations of female appetite. TV adverts which sexualise the eating of ‘naughty’ foods for women. Internet memes.
3. Emotion, anger and femininity Are there gendered expectations surrounding women expressing emotion, anger or rage? To what extent are women expected to sublimate their feelings and just ‘cope’ in a way that men - perhaps – are not? What are the political implications of this?	Representations of Elsa from *Frozen* (2013), including the song/sequence ‘Let it Go’. Evidence of readings made of Elsa online in relation to EDs, emotion, sexuality and more.
4. Reflection week on what we have done so far	
5. Reading the female body (1) What meanings might the thin female body convey? How does this relate to the idea of the starved female body? Can it only be read as an attempt to ‘over’ conform to the slender female ideal, or are there other ways of interpreting what might be communicated here? What does it mean to ‘take up space’, and how might these meanings be gendered?	Various media images
6. Reading the female body (2) As above	The participants read excerpts from other women’s’ narratives about why they think they developed an ED. They were then asked to situate these in relation to the groups so far.
7. ‘Healthy’ eating cultures and gender How does the exhortation of ‘healthy eating’ – and the cultural anxiety around the dangers of obesity – impact people with an ED? To what extent are media and public health messages about ‘healthy living’ and ‘healthy weight’ gendered, and still tied to a narrow range of ideal body images?	‘Healthy’ eating blogs, clips on ‘clean eating’
8. Fitness cultures and gender How is the contemporary ideal of the ‘fit’ body gendered? To what extent is the fit – as opposed to ‘just skinny’ – body still tied to a narrow range of body types and potentially oppressive self-surveillance/regulation practices? What do we think about the rise of ‘Athleisure’ wear, and what does it say about the relationship between body, fitness and gender?	# Fitspiration images from Instagram, Fitbit adverts
9–10 – reflections on the group, how it might relate to ‘us’, and how we might use its content in recovery	

This would sometimes involve them looking at images or watching clips as preparation for the group

In terms of awareness of power relations in therapeutic/treatment (as well as research) contexts, feminist approaches have increasingly acknowledged that relations between women are not necessarily ‘non-hierarchical’ (as simply based on gender congruence and perceptions of shared experience) [29], and that a range of factors mitigate against truly equal or collective dynamics. Although participants would shape the direction and breadth of the conversation, the facilitators set the broad agenda for each week, and the idea of researching a topic and ‘running’ a group clearly implies authority and expertise. Nevertheless, in seeking to invest in feminist models which reduce the gap between ‘expert’ and research subject – a perspective also key to feminist therapies - the facilitators participated fully in the group, regularly reflecting on their own relationships with the gendered discourses and ideologies being discussed, and sharing these with the participants.

### Measures

Sociocultural approaches to EDs have often drawn on a range of standardized self-report measures which examine and measure attitudes toward eating, body and exercise [7, 9, 10]. In comparison, feminist research has been more critical of such scales [5] – in large part because it sees EDs as the product of much wider discourses of gender inequity, and the standardized self-report measures tend to place a particular emphasis on eating behaviours and body image. As a result, feminist research has often (although not exclusively) used qualitative interviews, as commensurate with the feminist emphasis on listening to individual women’s’ experiences and views [30]. As the group undertaken here was exploratory, and the aim was to gather the participants’ general views on the possibilities of this treatment intervention, the primary method of data collection was the semi-structured individual interview. This is in turn based on the suggestion that making experiences and voices ‘visible’ (in this case, women’s views on an aspect of ED treatment), is a legitimate and important form of evidence in its own right [30].

The individual interviews were undertaken at the clinic with each participant 2 weeks after the groups had finished. These were conducted by the third-named author of this article who was not connected to the design or delivery of the groups in any way in order to increase the probability of participants offering honest appraisals of the group. The concept of *group* exploration had been central to the process and possibilities of the research (and the final session involved reflection on the groups in a group context). But it was hoped that individual interviews would allow participants the opportunity to express their own experience of the groups more fully. The interviews lasted for between 15 and 30 min, and participants signed a separate consent form beforehand agreeing for their data to be used in anonymised terms. The semi-structured aspect of the interview schedule then facilitated discussion of 5 key areas including: (1) how participants experienced the group and what they perceived were its key themes; (2) whether they felt that covering such themes had been useful/not useful to them as part of treatment; (3) whether the groups had challenged the participants’ understanding of why they developed or maintained their ED; (4) the implications (if any) of focusing on EDs as a product of society as opposed to a largely ‘individual’ problem; and (5) what the term ‘feminism’ meant to the participants, whether this understanding was changed or solidified by the groups, and what they thought about feminist approaches to EDs.

### Data analysis

The interviews were recorded on a digital voice recorder and then transcribed and anonymised by a research assistant. The data was then analysed using thematic analysis, an approach that seeks to identify, analyse and report patterns within qualitative data [31]. More specifically, the approach adopted here draws on thematic discourse analysis. Discourse analysis covers a range of language-orientated approaches, but is particularly concerned with how language actively constructs ‘reality’ within wider relations of power [32]. Although feminist research often seeks to make participant narratives visible and bring them into dialogue with political issues, qualitative studies do not simply or easily give participants ‘voice’ [13]. Indeed, discourse analysis involves construction and examination of the ‘underlying ideas, assumptions, and … ideologies that are theorized as shaping or informing’ what is said [31]. So in relation to the study here, exploring how the participants responded to the groups involved situating their comments in relation to broader frameworks of power which construct medical explanations of AN as having more authority and legitimacy than cultural or feminist interpretations, as well as examining the ideological contexts which might shape their interpretations of ‘feminism’ as a concept.

The data was approached using the six-stage process for thematic analysis defined by Braun and Clarke [31]. The first stage involved the first three authors familiarizing themselves with the data, so the transcripts were read and re-read independently so as to produce separate notes on preliminary ideas and observations. Second, this process was used by each of the first three authors to generate initial codes across the full data set (with two examples being ‘media and EDs’ and ‘responses to talking as a group’). Third, these initial codes were then checked and cross-checked between the three authors so as to generate broader thematic categories, with the key criteria being the prevalence of such themes within the data as a whole. In this regard, examples of thematic categories independently identified by the authors were questioning ‘normal’ ideas about gender, and ‘understandings of feminism’. Fourth, the themes were then tested against the coded extracts as well as the transcripts in their entirety. In stage five, the four themes that were identified inductively [31] were defined as (1) the privileging of ‘media blaming’ [33] discourses on EDs as representative of cultural factors in general; (2) feminist approaches to EDs as offering a form of ‘protective’ barrier to cultural messages during recovery; (3) tensions between sociocultural (e.g., feminist) and individualised (e.g., medical) understandings of EDs, and their implications for the participants’ conceptions of agency in recovery; (4) ambivalent conceptions of ‘feminism’. In step six, the thematic categories were then analysed in detail and data extracts which represented these themes (as well as the complexities and contradictions within them) were selected for inclusion. The writing stage then involved placing these themes in relation to the research questions and the literature on feminist approaches to ED treatment, and the divergences between the feminist and medical models of AN. Each of the four themes identified above are considered below as a way of exploring the responses of the participants and their implications for conceptions of ED treatment.

## Results and Discussion

### The availability and troubling of media blaming

Whilst remaining cognizant of the ways in which media constructions of the thin ideal are implicated within the aetiology and maintenance of eating problems [6, 7], the framing and content of the group aimed to explore the ways in which sociocultural factors in EDs may not be restricted to the effects of media images – thus engaging critically with the characterisation of EDs as the logical extension of ‘body image’ problems. It sought to do this openly and explicitly as part of the discussions, and participants were asked to consider why ‘culture’ might often be reduced to ‘media’ in constructions of AN, what their views were on this, and how this made them feel. Yet this attempt to problematize these discourses was not consistently foregrounded by the participants in the interview data, as they often returned to the centrality of ‘media blaming’ [31] discourses about EDs in various ways. This section seeks to explore these responses, and to contextualise their articulation and implications for thinking about the use of feminist perspectives on EDs in treatment.

The fact that those diagnosed with AN are encouraged to understand their problem as in part a direct response to media culture, and that culture is often reduced to ‘media’ in both popular and medical discourse, is suggested by the ways in which the power of media representations was immediately mentioned by the participants in interview. As one participant explained in describing what they saw as the key themes of the group: ‘*Obviously* a lot of it was around the media and how … that affected people with an eating disorder [our emphasis]’ (P1). But this was then usually followed up with a bid to question or complicate such a perspective (thus developing discussions that had happened in the groups). As one participant suggested:

So [it is] less like, well there’s a model, a skinny model in a magazine, looking at that, you’ve been looking at that too much and so you just wanna’ be like them … I don’t agree with that at all. I think that completely trivialises it (P7).

These responses support research which suggests that conceptions of AN as media-induced are perceived as fostering trivialising and stigmatising attitudes toward the problem (whereas knowledge of biological/genetic explanations are seen as decreasing such stigma) [34, 35]. As such research has shown, unlike contemporary ED treatment, public perceptions of AN have often foregrounded sociocultural explanations of eating problems which centre the power of the media – and related spheres such as the fashion and celebrity industries – in the production and maintenance of body/eating distress. Even though ‘individuals … are no more to blame for having sociocultural risk factors than they are for having biological risk factors’ [36], sociocultural factors are somehow perceived as more controllable, fostering a kind of volitional stigma in which an ED is trivialised as a behavioural choice [34, 35]. Empirical research has demonstrated how people living with an ED are highly aware of such assumptions and the stigma they carry, and that they may both critique and internalise such constructions in complex ways [35]. The participants in this study certainly critiqued the idea that mediated images of the thin ideal had produced their eating problem, but this was often framed as a response *to* the group – as if it the *group* had sought to convince them that this was indeed so.

As discussed in the methodology section of this article, the group *did* use media texts to stimulate discussion about the issues being explored: an example here would be the internet meme ‘Women Laughing Salad’ (a meme comprised of stock images depicting countless women ‘laughing with salad’) as a means to explore constructions of female ‘appetite’ (Table 1). Such media constructions were *not* framed or offered by the facilitators as ‘causal’ factors in AN (i.e. did images such as these make you ‘ill’?), but were rather situated as examples of broader cultural discourses on the aspect of femininity being explored. Nevertheless, the use of such texts may have shaped the emphasis on media culture in the interview responses.

But there may be more at stake here than the perceivably trivialising nature of media blaming discourses, as the participant responses may shed light on reactions to causal conceptions of AN more widely. The dominant discourse of the biopsychosocial foregrounds and privileges questions of causality – operating under the assumption that in order to treat (or ‘cure’) EDs, we must clearly understand their causes. Extensive research has been done on each aspect of the biopsychosocial equation in this regard [1], whilst the bid to pinpoint causal factors is also a dominant discourse in popular/media conceptions of EDs (particularly with regard to AN). Whilst the simplicity of media ‘effects’ explanations were critiqued in the group discussions, the group structure and facilitation also – at times – endorsed a causal model of AN (such as with Week 1, which included the discussion question ‘what causes EDs?’, albeit with a particular focus on how ‘culture’ is framed in these debates and constructions) (Table 1). As such, the participant above who refers to the ‘trivialisation’ of her experience may be responding to – and rejecting - wider models of liner *causality* as much the specificity of media-blaming discourses.

It remains striking, however, that only three participants explicitly described the focus of the group as being wider than media/body image, outlining the key themes as ‘looking at gender and also looking at different cultural beliefs about eating, appetite and … looking at different things that contribute to the eating disorder’ (P3), examining the ‘expectations on women in society’ (P4), or exploring the social construction of masculinity and femininity (P7). As one of these participants reflected:

I think [the group is about]... the deeper … more subtle factors that exist every single day since the day you were born … that you [are]… not really aware of, it’s just what society thinks and… everyone kind of adopts those kind of ideas [about gender], without even knowing… [The groups] have …contributed … to what I would have considered as society [sic] and cultural factors before. So I think before I saw that as like media and TV, magazines, whereas now I kinda see culture more as like the tiny little messages that you constantly get (P7).

But the fact that only some participants foregrounded what the facilitators envisaged as a key aim of the group does not suggest that the others had ‘misunderstood’ its focus. Rather, the responses may be seen as illustrating the power of the discursive frame which reduces sociocultural factors in EDs to questions of media, as well as the difficulty of seeking to challenge this ‘truth’ within a singular weekly context. In addition, it may also suggest a bid to the resist the privileging of causality in biopsychosocial models of AN, and point to the inadequacy of such paradigms in capturing personal conceptions of aetiology and experience. These contexts are clearly important in thinking about the challenges involved in introducing a group examining sociocultural aspects of EDs: existing explanations in this regard may already be seen as shallow or demeaning by the participants, and this may then effect group engagement and response.

### Feminist approaches to eating disorders offering a form of ‘protective’ barrier

In suggesting whether the focus of the groups should be part of ED treatment, all but one of the participants (who was unsure) felt that they should, and all said that they found the groups helpful to differing degrees. Emphasised here was the collective nature of the group, and its focus on recognising and questioning cultural and gendered constructions of food, eating, body and ‘healthy living’. Some of the responses spoke about the groups in ways which emphasised the general benefits of group work, including the discussion of shared experience, consensual validation, and a reduction in feelings of isolation [26, 27]. But the group context was also described as important in terms of the *specific focus* of the sessions - recognising experiences and beliefs as socially and culturally shared rather than the particular product of the individual. As one participant explained:

…if you’re with a load of different people … with lots of different upbringings, if you all think the same thing about certain things, there must be a reason why that is and then you can discuss whether that’s … cultural … So I think that’s better done in groups (P4).

From the point of view of the participants, these perceived benefits were sometimes, but not always, enmeshed within broader social/cultural expectations surrounding femininity. For example, although the concepts of media and body image often came up in the participant summaries of the group, the topics they then foregrounded as the most helpful were actually those which focused the *least* on exploring the body visually, such as gendered discourses on appetite in society, or discursive constructions of ‘healthy’ eating. Referring to the first topic, one participant explained how:

Because I think I’ve always just seen it as, oh, women have small appetites that’s just how it is or … like eating like this is ‘greedy’, that’s just how it is. But when you kind of see where that view comes from or how you’ve just been told that from like a young age it kinda’ makes you think *oh,* maybe that isn’t true [original emphasis] (P5).

This suggests that the participant is actively questioning what she had previously seen as ‘facts’ or ‘truths’ about femininity (‘oh maybe that isn’t true’), thus acknowledging such discourses as cultural constructions. This response, along with others in the study, articulated a key value of the group as fostering a greater literacy in reading cultural discourses surrounding body, gender and appetite – a recognition which does *not* automatically endorse the idea that people diagnosed with AN occupy a ‘heightened state’ of susceptibility [36] to such messages in the first place. There were different ways in which this literacy was developed in and fostered by the group, but as the response from P4 above suggests, a crucial element of this was the group context and how this offered the possibility of recognising ‘personal’ views as cultural constructions. So in the aforementioned week on appetite for example, some of the participants shared past stories of eating out with boyfriends and how they had felt compelled to eat less, or conversely, how boyfriends commented on food portions in ways which framed them as ‘excessive’ (for a girl). These feelings and reactions were then framed in a new light in the group when commonalities between participants/facilitators were established and heard.

In terms of functioning to offer a form of ‘protection’ from such ideological constructions, some participants felt that the group would also work as a preventative intervention, and with its emphasis on sociocultural ideologies surrounding gender, it indeed finds it closest companion in certain strands of preventative work [7]. At the same time, existing preventative programmes have been seen as engaging with the risk factor of gender in limited ways, thus privileging the concept of body image at the expense of other aspects of gender inequity [37]. Indeed, the response offered by P5 above suggests the potential value of expanding this focus (whether in preventative or clinical settings) into ‘gender-related contexts’ [37], such as the social and sexual regulation of appetite.

In reflecting on the extent to which the groups had been useful or otherwise as part of their treatment, the participants tended to suggest that the value lay less in them reappraising the aetiology of their ED (although some did feel that their conception of the role played by culture in this regard had increased), and more in terms of creating a protective framework in recovery:

It’s almost like, these social influences … are likely to be there… [so how can I] think about it rationally and ask like ‘what’s my actual view on that, why do people do that? How can I protect myself against that?’ So, yeah, I do think it can be [helpful] (P7).

In making this claim, the participant again acknowledges the potential importance of questioning normative values (‘what’s my actual view on that?’), and then positions this distance as a form of safeguard or defence against the ED itself. In this regard, the groups did not engender an epistemological shift in terms of how the participants viewed the aetiology of their eating problems or their sense of what an ED ‘was’ [4]. Rather, the main value of the sessions was articulated by participants in terms of developing a more critical attitude toward existing social constructs in ways not dissimilar to preventative interventions (even though the content and approach of the groups aimed to differ).

### Tensions between feminist and individualised understandings of eating disorders

As discussed earlier in the article, biomedical models of AN have been seen as endorsing individualised perspectives in which the ‘the person … is seen to have character flaws that need reparation, for example the person is emotionally unbalanced, needy, a perfectionist, desires control or has cognitive distortions’ [38]. In contrast, feminist approaches suggest that eating/body distress is situated within the context of normative femininities, and thus wider social constructions of women’s’ bodies, appetites and social roles. But different aetiological models of EDs necessarily impact upon participant conceptions of recovery: how likely recovery is, how it might be achieved, and on what terms [34, 35]. In this regard, aetiological models which place the focus on sociocultural contexts may pose complex questions. This is especially so in terms of agency, ‘blame’ and the possibilities of recovery, particularly when participants are more accustomed - in treatment contexts at least - to having their problem framed in individual terms.

As one participant reflected:

… I think at first I did just think okay, maybe this [ED]… is all down to me, and I always kind of dismissed the idea as ‘oh society doesn’t really play a part’. But then, as the groups went on its like okay, maybe this society’s norms are quite disordered. But then it’s like … if society’s norms are disordered … then … I don’t know, how am I meant to change kind of thing? (P5).

The response suggests that the groups may have played a role in decreasing feelings of self-blame (as indicated by the questioning of ‘maybe this is all down to me’). From a feminist point of view, this is potentially significant, as it has been suggested that women are often socialised to internalise the blame for problems that emerge from structural gender inequalities [39]. At the same time, confronting the possibility of the latter appears to raise difficult questions about how to fully recover (‘how am I meant to change kind of thing?’) if the locus of ‘disorder’ is not so firmly rooted in the individual. This is particularly salient to understandings of AN given that issues of control and self-control have often been foregrounded as core drivers and themes [40]. Furthermore, although investing in cultural perspectives on EDs may mitigate feelings of self-blame, putting EDs on a continuum with *normative* social values (or ideologies) could end up confirming, for some participants at least, the same pathologising logic that the feminist approaches claim to avoid:

The only … question… in my head [is] that so many people are faced with these messages from society, but that, for some reason I‘m really affected by them …. [L]ike why am I really affected by them if everyone has the same messages? (P2).

The fact that not *all* women develop such problems acutely has been the aspect of the feminist scholarship on EDs most attacked by the biomedical model [3, 6], and it returns to what is seen (by both participants and the feminist perspectives) as the problematic spectre of the ‘vulnerable’ media consumer. Such biomedical criticisms of the sociocultural/feminist approaches have been addressed eloquently elsewhere [3, 6]. But it is clear in the response above that feminist perspectives may not always mitigate the feelings of ‘difference’ or ‘deviance’ that structure the experience of being diagnosed with an ED, and we can see the participant working through where to situate herself – and her ‘AN’ identity - in relation to sociocultural explanations of EDs.

The quote may also be seen as indicative of how the groups did not fundamentally challenge the participants’ views of what an ‘ED’ was (they were often reluctant to relinquish the biomedical claim to AN as an ‘illness’ for example), and thus radically alter their positioning (s) in this regard. Notably, this was in contrast to a previous study undertaken by one of the authors [4] in which a sample of women who had recovered from an ED were encouraged to consider the feminist approaches, and to reflect upon their implications for how they had conceptualised their problems with eating. This study suggested the possibility of the feminist approaches effecting an epistemological *shift* in this regard – or what one participant in the research referred to as a ‘real lightbulb moment’ which ‘changed her life’, as well as her conception of what AN ‘was’ [4]. Why this wasn’t the case for the participants in the clinic groups is complex: it may have been shaped by the participants’ ambivalent relationships with feminism (see below); the fact that they were living with very acute eating problems and were in the earlier stages of treatment; or that some held very clear views on why they had developed an ED (having lived with AN for many years). The disparity here may also have been shaped by the facilitation of the group, which was careful about how the feminist challenge to the medical model was framed. After all, it would not have felt ethical or appropriate to encourage the patients to openly critique the biomedical paradigm when it was otherwise structuring much of their current treatment (and such a critique may not have been accepted by the clinic). In this regard, the feminist perspectives were often presented as offering a ‘further’ perspective on EDs, returning us to Sampson’s notion of accommodation vs transformation [54] and how the ‘new’ is ‘added’ to the old without fundamentally challenging its dominant truths and hierarchies. This then also raises further questions - germane to feminist knowledges and therapies more broadly - about how far, and on what terms, feminist approaches to EDs can work alongside dominant biomedical paradigms when they critique their fundamental principles.

### Ambivalent reactions to feminism

As noted, the responses to the group were also inevitably shaped by the participants’ relations with feminism - something which emerged as an ambivalent concept for many of the participants in the study. Although one of the group facilitators identified overtly as a feminist, both facilitators were aware that the term could carry negative ‘baggage’ which may impact upon group engagement. As such, it was decided not to persistently label the focus the groups as ‘feminist’, even though they clearly and openly engaged with the cultural construction of femininity. The groups themselves did not constantly invoke the terms ‘feminist’ or ‘feminism’, but the interviews asked the participants about their views on these concepts, and whether they saw the group as ‘feminist’ in approach. This question was included because the research wanted to consider the extent to which participants’ views on gender politics may have shaped their responses to the groups. But making these questions explicit prior to the groups (rather than after) may have set up particular expectations of and resistances to the groups that the facilitators were keen to avoid.

As different scholars have explored over the last 10 years, ‘feminism’ stands as a fraught concept in an era in which young women in particular are perceived to be disaffected from feminist politics, as shaped by discourses of generationalism, post–feminism and neoliberal ideologies of individualism and choice [41, 42]. It has recently been suggested that we are seeing signs of change, and entering a political and cultural moment in which ‘feminism has moved from being a derided and repudiated identity among young women… to becoming a desirable, stylish and decidedly fashionable one’ [43]. Nevertheless, the data presented here is consistent with the proposition that the ‘repudiation’ of feminism continues in complex ways.

The responses given in the interviews often exemplified the position described by Rich in her discussion of how (young) women negotiate feminism within a neoliberal framework: the tendency to invoke and support an ‘equal opportunities framework yet also distance themselves from feminism, and in particular, the subjectivity of “feminist”’ [42]. As one participant reflected:

Feminism…. I like the idea of women having rights… but then I think it can also go borderline, you know, women are great, men aren’t… When we ask for more and more and more through feminism, I think it’s borderline sexist (P2).

Feminism emerges here as appropriate and sanctioned in terms of ‘women having rights’. But in the suggestion of asking for ‘more and more’ it is implied that such ‘demands’ are ‘aggressive, unfeminine, and in some ways unacceptable’ [42] – a perspective that, like AN, is itself bound up with ideas about what constitutes ‘appropriate/inappropriate’ expressions of femininity [3]. None of the participants suggested in their interviews that they identified *as* feminist, and their responses moved across a continuum from relative interest, clear disinterest, to outright rejection of the concept. In outlining this, the research is *not* endorsing the problematic idea that people diagnosed with AN are more likely to be aligned with patriarchal and subordinated modes of femininity, and should somehow be positioned as the antithesis to a ‘feminist’ identity [44]. Such generalisations are problematic and potentially disempowering, and feminist work has long since explored AN as a potential resistance to or rejection of normative femininity, and always a complex negotiation of its discursive power. Furthermore, and as acknowledged above, the participants’ reactions to feminism can also be situated within a particular historical and political context in which the concept has highly contested meanings, and the groups cannot exist outside of this context. In this regard, their attitudes are less specific or ‘aberrant’ than they understandable and ‘normal’ for many women today.

All of the participants suggested that they perceived the groups to be feminist to some degree, and they were asked in the interview what this term meant to them in the context of sessions. While some suggested that ‘it’s kind of the way that there are different expectations of men and women around food… and weight loss’ (P7), others felt that the gender focus was extreme:

People that aren’t female get eating disorders as well… I think that men are also held to quite high standards when it comes to exercise and things like that so…I don’t know, I don’t think eating disorders really are a feminist issue (P4).

The question of whether the feminist approaches can be applied to boys/men with EDs is an important one in debates about gender-inclusive treatment [45], and potential disparities here – as well as the rising pressures on men in terms of fitness cultures and body surveillance - were often discussed in the group itself. But in the response above, there appears to be an anxiety about feminism disempowering males with EDs (individuals with whom the participants may feel solidarity given the typically isolating and stigmatising consequences of an ‘anorexic’ identity), as well as a rejection of girls/women as the specific ‘victims’ of patriarchy. At the same time, there were contradictions in terms of how feminist perspectives were evaluated in the interviews: for example, although the weeks in which the group examined gendered constructions of appetite were often cited as the most ‘feminist’ or explicitly gendered, it was also these that were highlighted as the most productive and helpful, as responses used earlier in the article suggests. This may suggest a split between how the identity of the feminist is perceived (as the figure of the ‘man-hating … unfeminine’ woman [42]), and then how feminist ideas are then experienced in practice.

## Conclusions and implications

This study has explored responses to a 10-week group trial based on feminist approaches to EDs with a group of seven female patients. Compared to existing conceptions of sociocultural factors in EDs (which can be found in sociocultural paradigms, body image treatment, preventative work and popular media discourse), the group sought to adopt a broader perspective on how gender/culture might be approached in relation to eating/body distress, primarily drawing on feminist research. In doing so, the research aimed to address two important questions: To what extent were a series of groups on the relationship (s) between EDs and cultural constructions of femininity seen as useful and beneficial to the participants? How did they respond to a perspective which sees EDs as primarily sociocultural, rather than individual, manifestations, and what were the implications of this for how they conceptualised their ED and the possibilities of recovery?

The participants suggested that the groups were helpful in enabling them to situate their problem within a broader social/cultural and group context; that they could operate as a form of ‘protection’ from dominant ideologies regarding female corporeality, identity and appetite, and that they could potentially reduce feelings of self-blame. Furthermore, the data also offers some support for the idea that focusing on broader gendered discourses – such as those relating to appetite for example – might be productive for participants in thinking about how often unquestioned (and potentially more ‘invisible’) gender inequities may shape the aetiology and maintenance of an ED.

But focusing on the interaction between the individual and the social also had contradictory implications for the participants (and yielded contradictory results). First, although the participants were highly critical of the ways in which ‘culture’ is often reduced to mediated images of (‘ideal’) bodies in discussions about AN, it was also difficult to decentralise this equation in their expectations of, and responses to, the group. This suggests that existing discursive frames relating to how ‘culture’ is conceptualised in relation to EDs (and AN in particular) may play a significant - and unavoidable - role in how participants respond to treatment interventions which take a sociocultural stance. That said, the article has also considered how such responses read more positively in terms of resistance to the *causal* emphasis of biomedical paradigms, and the ways in which these simplify the lived experience of an ED. Second, the feminist approaches raised potentially unanswered questions for the participants about how different aetiological models of EDs may impact upon perceptions of recovery. Although feminist approaches to EDs have seen medical and biological conceptions of EDs as pathologising and stigmatising, locating the ‘problem’ within the self may nevertheless offer the individual a greater sense of personal agency than societal frameworks when it comes to conceptions of recovery. In this regard, the feminist frameworks were clearly dilemmatic for the participants, offering ways to situate and critique what they had seen as ‘personal’ messages about femininity within a group context, but also challenging feelings of agency and autonomy. Third, the responses of the participants suggest that the feminist bid to place AN on a continuum with normative femininities may itself foster aspects of social and cultural stigma. In this regard, their responses sometimes echoed the common biomedical critique of feminist/sociocultural work on EDs which asks ‘If the “cultural rain” is so powerful, why is it only a relatively few girls and women “get soaked” and proceed to develop eating disorders?’ [6]. Finally, although the emphasis on thinking through the potential links between AN and cultural constructions of femininity was often received positively by the participants (encouraging them to question ideologies and beliefs about gender which they had taken for granted), the idea of ‘feminism’ emerged as an ambivalent and contested concept in the interviews, thus shaping both participant engagement and response.

There are clearly limitations to the sample and the data, and these must be considered here. Although the aim of the study was not to generalise findings to *all* women who are treated for AN (but rather to consider the efficacy of the feminist groups for these particular participants), the sample size (*N =* 7) is clearly small. The time-span of the groups (10 weeks), is also limited in the context of group work and the possibilities of group functioning, and the implementation of follow-up measures would elucidate any longer-term implications of the groups beyond the data collected here. Furthermore, the treatment setting meant that the participants had experience of chronic and/or long-term AN: the results may be different in an out-patient setting; with people who were further along in recovery; with participants whose symptoms were less severe; or with participants who had different conceptions of gender politics and feminism. In addition, feminism, and feminist work on EDs, has historically been critiqued for foregrounding gender inequalities at the expense of other hierarchical systems of power – such as ethnicity, sexuality and class. The authors fully recognise the intersectional nature of gender identity, and do not see femininity as a homogenous construct. But it remains the case that the groups tended to focus on gender as a concept (with only marginal space given to factors such as ethnicity and class), and that the voices in this study are primarily white - as with the bulk of feminist research on EDs [4]. Finally, there were also limitations to how openly and explicitly the feminist approaches could be set up as a critique of biomedical paradigms in the context of the clinic groups. This may have reduced the possibility of the groups really challenging how the participants saw their EDs, and thus their identities as ‘eating disordered’ women.

Nevertheless, the results of the study, whilst mixed and complex, have implications for understandings of contemporary ED treatment, and the role of feminist perspectives within this context. There is substantial evidence that EDs *are* imbricated within, and shaped by, sociocultural contexts [6], whether in terms of risk, aetiology and maintenance, and the prevalence of girls/women in ED populations is the most ‘obvious’ evidence of this. But such issues are often positioned as secondary or contextualising factors within treatment in ways which are individualising and problematic. It is important in this regard to think about the status and nature of ‘evidence’ that is required for treatment practices to develop. Contemporary conceptions of evidence-based ED treatment favour Cognitive Behavioural Therapy (CBT) because this is where the best evidence current base currently lies [2, 46]. But these results also reflect the fact that CBT has been the *most researched* approach in the treatment of EDs. Other approaches cannot acquire an evidence base unless they are tested and explored. Although this would evidently need to be on a *far* larger scale than the study mounted here, it may be important to consider that different approaches and epistemologies produce different types of evidence: not all approaches are necessarily amenable to the scientific measure of the ‘gold standard’ randomised clinical trial, and feminist perspectives have often invested in qualitative methodologies which are critical of ‘objectivist’ views of science and evidence [18]. These too can play a valuable role in elucidating the shortcomings and limitations of contemporary ED treatment.

Second, in terms of future work, it is clearly important to test out the feminist approaches with different samples, in different contexts, and in different ways. Any such endeavor will need to consider how ‘reactivating’ these approaches requires negotiation with the complex cultural status of feminism today. It will also need to consider how the wider context of biomedical ED treatment may limit what it is possible to ‘say’ and achieve with the feminist approaches. In terms of the fact that the groups did not necessarily enable participants to shift their position (s) with regard to their conception of, and relationship with, their ED, it may be that drawing on narrative therapy [47] as a therapeutic ingredient would be useful here. As with the feminist approaches, narrative therapy seeks to externalise AN away from the individual, open up alternative ways of positioning the self in relation to the ‘problem’, and generate new self-understandings [48, 49]. In so doing, it too is critical of the objectivity of medical knowledge, and with regard to gender politics, it seeks to provide the individual with alternative perspectives on dominant gender scripts and to ‘politicize these in the context of larger cultural narratives’ [50]. But, in returning to the idea of the accommodative vs transformative (Sampson), concerns have also been raised about how easily narrative therapy can be inserted into biomedical interventions [50], and such an approach is far from mainstream in the US/UK. Along with feminist conceptions of EDs, it remains labelled an ‘alternative’ approach to ED treatment – suggesting the existing power of biomedical treatment models.

Third, it is important to stress that the study presented here does not work on the assumption that issues concerning gender identity are only relevant to the experience and treatment of EDs in girls/women. The vast majority of feminist work on EDs has indeed concentrated on this patient group. But the focus on how eating/body distress may be used to negotiate dominant ideologies concerning gender/sexuality is similarly applicable to male patients [51], as well as sexual [52] and gender minorities [53], even whilst the cultural constructions at stake may be different [54]. Discussions of EDs in the male population have led to calls to ‘tailor’ treatment, including perspectives on sociocultural concerns, to boys/men [51, 54, 55]. It is clearly imperative that ED treatment is gender inclusive (for both males and gender minorities), and qualitative research with male patients has highlighted the difficulty faced in negotiating the ‘female character of the services’ - in terms of the lack of gender-appropriate assessment criteria [56], or treatment environments which are largely occupied by females [55]. But the suggestion in such discussions that male patients are left with ‘no gender-specific techniques that distinctively address the psychosocial aspects of their disorder’ [51], or the insistence that ‘treatment paradigms have been *geared toward* females [emphasis added]’ [55], may be misleading in assuming that *female patients are well served in this regard.* There is little or no evidence that contemporary ED treatment focuses on the relationship between EDs and cultural constructions of femininity as a systematic or sustained aspect of clinical work, despite the clear and continued prevalence of cis-gendered female patients [4]. Given the substantial empirical base which suggests that ED are culture-bound on a range of different levels [1, 6], this omission seems deeply problematic.

Finally, the study has implications for how sociocultural factors in EDs are conceptualised: by clinicians, patients and the general public. The research suggests that how sociocultural factors in EDs are perceived by patients (and how they *see* them as being perceived by others) may pose a barrier to exploring the relationship between eating problems and their cultural contexts. If patients do not perceive the sociocultural aspects of EDs to be treated seriously and sympathetically within popular and clinical understandings of their problem (and if they are not given space to explore the complexity of such issues themselves), this may hinder willingness to engage in such interventions when they are offered. Studies of stigma that explore clinician, popular or patient conceptions of EDs have tended to ‘urge policymakers and the general public to acknowledge the biological contributions of these illnesses’ [57] in a bid to de-stigmatise eating problems, whilst they also advocate for the importance of increasing ‘mental health literacy’ [35]. These are valid points. But rather than rejecting the possibility of sociocultural conceptions of EDs contributing to more complex and less stigmatized understandings of eating problems (which currently endorse gendered conceptions of EDs as ‘vain, trivial and voluntary’ [14]), it would seem pertinent to address and complicate the problematic ways in which sociocultural aspects of EDs are perceived.

## Abbreviations

(AN): Anorexia nervosa
(BED): Binge-eating disorder
(BN): Bulimia nervosa
(ED): Eating disorder
(NHS): National Health Service
(OSFED): Other specified feeding and eating disorder
